# Accelerating The Virtual Brain with code generation and GPU computing

**DOI:** 10.1186/1471-2202-14-S1-P198

**Published:** 2013-07-08

**Authors:** M Marmaduke Woodman, Viktor K Jirsa

**Affiliations:** 1 Institut de Neuroscience des Systemes UMR 1106, Aix-Marseille University, Marseille, 13005, France

## 

The genesis of both theory of neural field modeling and forward solutions for neuroimaging modalities and empirical methods to obtain connectivity datasets necessary to simulate the human brain dynamics has lead to the development of a whole-brain simulator, built within a freely available neuroinformatics platform called the Virtual Brain (TVB) [1-3]. TVB and the research that led to it, has opened up several possibilities as well as further research questions. On the one hand, such simulations reproduce aspects of human resting state activity, and on the other, it has been observed that certain neural pathologies manifest changes in the resting state, therefore suggesting that the Virtual Brain presents an opportunity to model the dynamics of a brain, on a case by case basis, enabled by non-invasive tractography methods such as diffusion tensor or spectrum imaging. Other use cases are also possible, e.g. in the case of stroke patients to model the effects of resulting lesions on resting state dynamics. However, obstacles remain: First, the relative effects of the parameters on the network model stability and its associated parameter dispersion in TVB are not well understood, and second, because of realism of finite conduction velocities in the brain connectivity in TVB, the models remain computationally expensive. In the work presented here, we address both obstacles in computational terms by introducing and implementing strategies to improve the performance of the simulator in TVB, based on generating customized code per simulation [4]. These improvements are drastic when access to recent graphics processing units (GPUs) is available and represent orders of magnitude increase in the ability of users to explore both parameter spaces or qualitative dynamical regimes, and therefore significantly amplify the impact of whole-brain modeling approaches.
